# Words and Their Uses

**Published:** 1885-11

**Authors:** 


### "Words and Their Uses."

We observe with great pleasure signs of progress in medical journalism in a recent issue of our esteemed contemporary, the *Journal of the American Medical Association.*

Attention is called in an editorial to certain "misused words." We hope to be pardoned if we presume to suggest that some reasons should be adduced, when an editor engages in verbal criticism, and advises changes in the use of terms. "Apt" is frequently written when "likely" should be employed. RICHARD GRANT WHITE says, with reference to the use of "apt," "This little word is in danger of losing its fine sense, and of being degraded into a servant of general utility for the range of thought between *liable* and *likely*." "Aptness and liability both express condition—one of fitness and readiness, the other of exposure—inherent in the person or thing of which they are predicated."

With reference to the use of the word "allude," we fully agree with our editorial brother. "*Allude* is not a fine sounding synonyme of *say* or *mention*." "*Allude* (from *ludo*, *ludere*, to play) means to indicate jocosely."

Great difference of opinion exists as to the use of the words "diagnose" and "diagnosticate." Etymology throws little light upon the subject. Usage undoubtedly favors the employment of "*diagnose*."

However, we are not disposed to be hypercritical, and cheerfully accept the editorial strictures, appearing in the *Journal of the American Medical Association*, as an augury of what that periodical is likely to be with reference to the correct use of English terms.

